# Multi-step depth enhancement refine network with multi-view stereo

**DOI:** 10.1371/journal.pone.0314418

**Published:** 2025-02-13

**Authors:** Yuxuan Ding, Kefeng Li, Guangyuan Zhang, Zhenfang Zhu, Peng Wang, Zhenfei Wang, Chen Fu, Guangchen Li, Ke Pan

**Affiliations:** 1 College of Information Science and Electrical Engineering, Shandong Jiaotong University, Jinan, Shandong, China; 2 Shandong Zhengyuan Yeda Environmental Technology Co., Ltd, Jinan, Shandong, China; University of the Punjab, PAKISTAN

## Abstract

This paper introduces an innovative multi-view stereo matching network—the Multi-Step Depth Enhancement Refine Network (MSDER-MVS), aimed at improving the accuracy and computational efficiency of high-resolution 3D reconstruction. The MSDER-MVS network leverages the potent capabilities of modern deep learning in conjunction with the geometric intuition of traditional 3D reconstruction techniques, with a particular focus on optimizing the quality of the depth map and the efficiency of the reconstruction process.Our key innovations include a dual-branch fusion structure and a Feature Pyramid Network (FPN) to effectively extract and integrate multi-scale features. With this approach, we construct depth maps progressively from coarse to fine, continuously improving depth prediction accuracy at each refinement stage. For cost volume construction, we employ a variance-based metric to integrate information from multiple perspectives, optimizing the consistency of the estimates. Moreover, we introduce a differentiable depth optimization process that iteratively enhances the quality of depth estimation using residuals and the Jacobian matrix, without the need for additional learnable parameters. This innovation significantly increases the network’s convergence rate and the fineness of depth prediction.Extensive experiments on the standard DTU dataset (Aanas H, 2016) show that MSDER-MVS surpasses current advanced methods in accuracy, completeness, and overall performance metrics. Particularly in scenarios rich in detail, our method more precisely recovers surface details and textures, demonstrating its effectiveness and superiority for practical applications.Overall, the MSDER-MVS network offers a robust solution for precise and efficient 3D scene reconstruction. Looking forward, we aim to extend this approach to more complex environments and larger-scale datasets, further enhancing the model’s generalization and real-time processing capabilities, and promoting the widespread deployment of multi-view stereo matching technology in practical applications.

## Introduction

Convolutional Neural Networks (CNNs) have been widely applied in computer vision tasks, offering superior performance in processing geometric structures’ correlation and spatial matching compared to 2D CNN methods [1–3]. Deep learning-based Multi-View Stereo (MVS) approaches mainly focus on improving the quality of reconstruction; however, the efficiency of reconstruction is an equally important aspect that cannot be overlooked [4]. For instance, FastMVSNet [5] enhances reconstruction efficiency by constructing sparse cost volumes and using small-scale CNNs to encode local pixel depths before filling in high-resolution depth maps. MVS-CRF [6] combines the predictive depth mapping of a learnable CRF model with the local feature extraction capabilities of deep learning within a global optimization framework, further increasing the accuracy and efficiency of depth estimation. The advancements in these techniques have propelled the development of MVS and its related technologies.

Our proposed Multi-Step Depth Enhancement Refine Network (MSDER-MVS) introduces innovations in the multi-view stereo field, particularly through its dual-branch fusion architecture. This architecture uniquely combines high-level semantic features with detailed spatial information from multi-scale feature maps, enabling more accurate depth estimation and efficient computation. The dual-branch design allows for the parallel extraction and integration of features from different image scales, effectively capturing both coarse and fine details in the scene.

Additionally, while Feature Pyramid Network (FPN) has been utilized in other MVS approaches, MSDER-MVS leverages an advanced implementation of FPN tailored for multi-view stereo tasks. This implementation enhances the model’s ability to handle high-resolution inputs by selectively integrating multi-scale features, thus optimizing both the precision and the speed of depth reconstruction. The integration of FPN with our dual-branch fusion approach results in a comprehensive feature representation that enhances the quality of the generated depth maps.

Furthermore, the network incorporates a multi-step depth refinement strategy, which iteratively refines the depth maps across multiple stages. This approach not only improves the initial depth estimates but also progressively enhances the fine details, leading to more accurate and complete 3D reconstructions. The unique combination of these architectural elements makes MSDER-MVS distinct in its capability to handle complex scenes with varying depth scales and detailed textures.

In a plethora of widely adopted studies, Yao et al. [7, 8] have proposed a method that employs a sophisticated plane sweeping process coupled with variance-based cost volume construction for each reference image to establish predictive depth maps. This approach is further refined by the utilization of multi-scale 3D convolutions to regularize the cost volume, thereby optimizing computational efficiency while maintaining accuracy [9]. R-MVSNet [10], an innovative framework, ingeniously leverages the capabilities of Recurrent Neural Networks (RNNs) [11] for sequential regularization of the cost volume. This method has not only achieved a reduction in data processing volume from cubic to quadratic scale but also substantially reduced the memory consumption associated with increased model resolution [12, 13]. This improvement not only elevates the efficiency of 3D reconstruction techniques but also makes possible the handling of high-resolution data, thereby broadening the potential and scope of 3D vision technology in real-world applications.

We have introduced a dual-branch fusion network designed to enhance the quality of depth maps by circumventing the burdensome 3D convolutions. This method, based on the extraction of image pyramids, captures multi-view features for stereoscopic matching. By constructing the cost volume from coarse to fine, the method initially focuses on a rough estimate of the low-resolution depth map, then at finer levels, narrows down the current depth hypothesis based on the low-resolution depth information. Moreover, we employ a multi-step depth enhancement refinement technique to increase the pixel-level precision of the depth maps. This optimization, integrated as part of the network, eliminates the need for additional learning parameters.

## Related work

In this chapter, we review research within the field of computer vision, particularly focusing on multi-view stereo (MVS) [7, 8, 10, 12, 14] reconstruction and depth estimation. Our discussion will center on the evolution from traditional methodologies to those based on deep learning, as well as how these approaches influence each other and continue to push the boundaries of technological development.

The development of multi-view stereo reconstruction has always been a core topic in computer vision research. Initially, researchers relied on traditional principles of photogrammetry [15–18], using images captured from different viewpoints to match feature points and build 3D point clouds. While these methods are theoretically sound, they are limited in practice by errors in feature matching and the sparse distribution of data [19]. With the advancement of depth cameras and structured light scanning technologies, researchers have been able to obtain richer three-dimensional geometric information from large-scale datasets, demonstrating great potential for depth estimation and precise reconstruction in complex scenes [20].

By integrating deep neural networks, revolutionary improvements have been made in the construction of cost volumes and depth prediction. MVSNet, developed by Yao et al. [7, 8], through end-to-end training, has learned the intrinsic rules of depth estimation from a vast number of samples, significantly enhancing the accuracy and stability of depth predictions. This learning-based approach has freed researchers from the complex adjustments of parameters and algorithm design, allowing them to focus on innovations in network structures and training strategies.

However, as the resolution of input images increases, the size of the cost volumes processed by MVS methods expands rapidly, leading to a surge in the demand for computational resources. This has prompted researchers to explore depth-optimized networks, such as FastMVSNet [5], which optimize computational efficiency through the construction of sparse cost volumes and efficient network architectures, and enhance the precision of depth estimation through iterative optimization strategies [21].

Moreover, the concept of cascaded cost volume [22–24] has been applied to variants of MVSNet. CasMVSNet [22] incrementally enhances the fineness of depth predictions by constructing the cost volume in multiple stages. This strategy reduces the direct computational demands for high-resolution depth information, adopting a coarse-to-fine approach to gradually refine depth details, effectively balancing accuracy and computational efficiency.

Recent advances in this field include the MVSTR network, which utilizes a global-context Transformer module and a 3D-consistency Transformer module to capture dense and globally consistent features for multi-view stereo, showing excellent performance on the DTU dataset. Additionally, the SSC-MVS framework introduces a novel unsupervised learning strategy with pseudo-depth supervision and a consistency-based training mechanism, achieving state-of-the-art results among unsupervised methods and demonstrating potential to outperform fully supervised approaches [25, 26].

The 2T-UNet model replaces traditional cost volume construction with a dual-tower convolutional neural network, utilizing left and right stereo images along with monocular depth cue information as inputs, significantly enhancing the quality of scene geometry prediction. It surpasses existing monocular and stereo depth estimation methods, particularly in complex natural scenes. Additionally, the deep learning architecture for multi-exposure stereo depth estimation introduces innovative stereo matching techniques and a mono-to-stereo transfer learning approach, avoiding traditional cost volume construction. By fusing disparity maps at different exposure levels, it provides robust support for various 3D HDR applications. Lastly, the SDE-DualENet model achieves stereo depth estimation without constructing a cost volume by employing a dual-tower convolutional neural network based on the EfficientNet architecture. This method performs pixel matching with different weights in the dual towers, excelling in handling complex scenes with high detail and large depth variations [27–30].

As the field of MVS progresses, researchers have also explored hybrid methods that combine traditional geometric techniques with deep learning. For example, the MVS-CRF [6] model enhances global consistency and detail recovery in depth estimation by integrating the feature extraction capabilities of convolutional neural networks with conditional random fields. These hybrid models leverage the automatic feature learning capabilities of deep learning and refine reconstruction results through geometry-driven global optimizations.

Against this backdrop, our research aims to explore and refine multi-view stereo matching techniques. Our proposed method, the Multi-Step Depth Enhancement Refine Network (MSDER-MVS), addresses the efficiency and accuracy issues of previous technologies when processing high-resolution data. By considering the depth and breadth of the network architecture and the diversity of optimization strategies, MSDER-MVS shows significant improvements in accuracy and efficiency.

Our network adopts a dual-branch fusion architecture, cleverly avoiding reliance on large-scale 3D convolutions, thus significantly reducing computational costs. Additionally, we fully utilize the concept of feature pyramids to capture rich multi-view information through multi-scale feature extraction, enhancing the network’s sensitivity to geometric details in images. Our method initially generates coarse depth maps at lower resolutions, then refines depth estimation at finer levels using a compact cascaded cost volume strategy. Not only does this strategy visually improve reconstruction quality, but it also optimizes efficiency while maintaining details.

Furthermore, our unique depth optimization method uses an efficient iterative computation strategy to enhance the accuracy of depth predictions while maintaining network feedback speed. This innovative approach in deep learning uses residual information and incremental calculations between feature maps to gradually refine the depth map. Our optimization process is differentiable, making it an integral part of the neural network, thus eliminating the need for additional learnable parameters.

In terms of loss function design, we employ a comprehensive multi-stage calculation method that not only considers the accuracy of depth estimation at each stage but also balances the results across different stages to ensure the consistency and reliability of the overall depth map. This approach allows our network to manage errors in both initial and refined depth estimates effectively, ensuring high-quality final depth maps.

In summary, our Multi-Step Depth Enhancement Refine Network represents a significant advancement in multi-view stereo matching technology. It not only demonstrates excellent performance on existing datasets but also lays the groundwork for future applications in high-quality 3D reconstruction.

## Method

**Fig 1:**
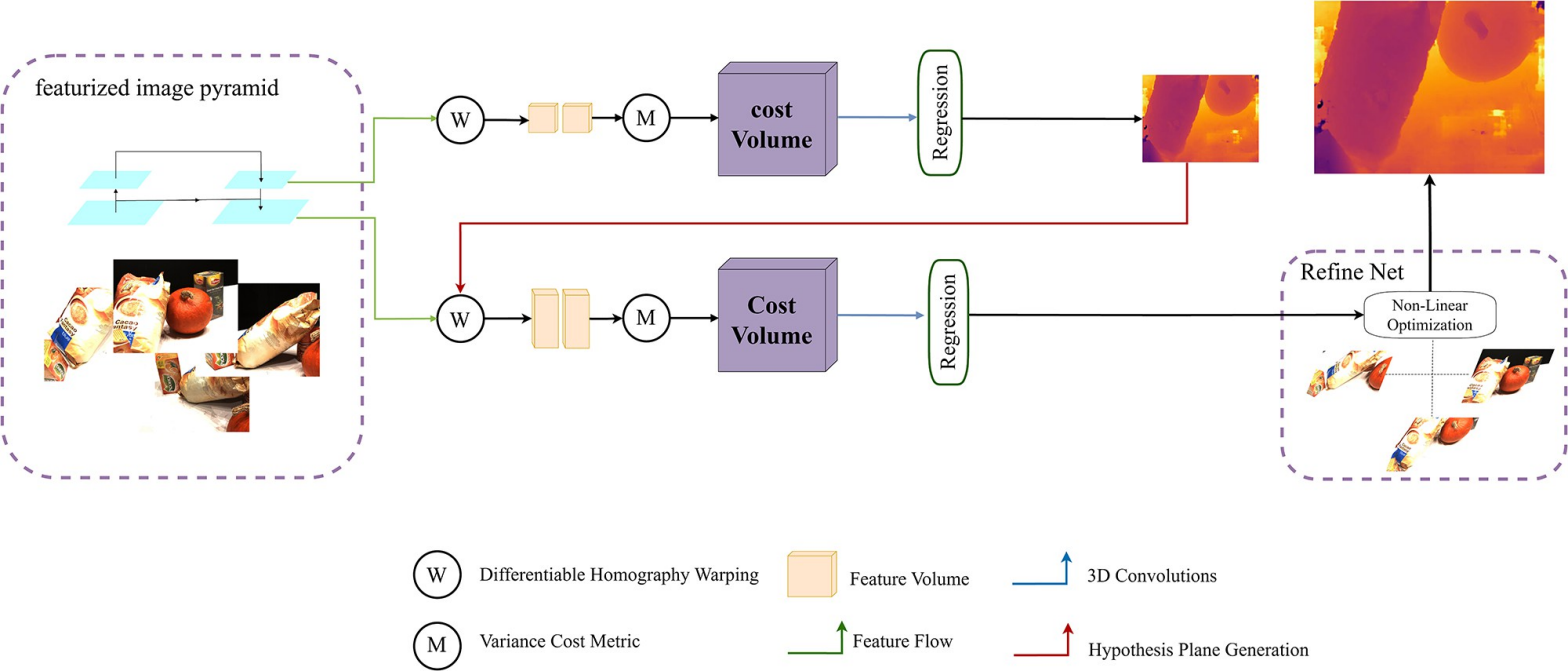
Network architecture of the network, denoted as MVSNet+Ours. Multi-Step Depth Enhancement Refine Network with Multi-view Stereo.

In this section, we detail the core contributions of our network, starting with existing multi-view stereo matching techniques. Our method initially generates a preliminary depth map at a lower resolution. Subsequently, through the application of a cascaded cost volume strategy, the depth for each view is refined progressively from coarse to fine. During the refinement phase of depth prediction, we employ a multi-step depth enhancement refinement method to enhance the detail in the depth map and further improve the pixel-level accuracy of the depth map. Fig 1 illustrates the overall architecture of our method, which combines meticulous hierarchical progression with efficient optimization algorithms, aimed at achieving higher quality depth reconstruction.

### Using feature pyramids for feature extraction

In our research, we employ a Feature Pyramid Network (FPN) [24] to extract features from multi-scale images for depth estimation. This approach leverages information at various resolutions, where a feature extraction network extracts feature maps rich in semantic information from each scale. Specifically, we designed a two-layer FPN to enhance computational efficiency while ensuring effective feature extraction.

In our FPN implementation, the first layer of feature maps originates directly from the base feature extraction network with a resolution of one-fourth the size of the input image. This layer captures high-level semantic information but may lack detailed information. To address this, we designed a second layer of the FPN that upsamples the feature maps from the first layer to the original size of the input image through convolution operations, thereby incorporating higher resolution spatial information. This design enables the second layer of the FPN to merge strong semantic information from low resolutions with rich spatial information at high resolutions, effectively capturing image features across different scales.

Furthermore, to optimize the feature fusion process, we introduced a transition layer between the two FPN layers, which converts the channel count of the feature maps from the first layer to that of the final output, ensuring consistency during feature fusion. Through this method, the transformed feature maps integrate better with high-resolution feature maps, thus enhancing the accuracy of depth estimation.

Ultimately, by adjusting different levels within the FPN, we constructed a cost volume corresponding to the input image sizes of 1/4, 1 in spatial resolution. This multi-scale feature fusion strategy not only integrates image features across different resolutions with reduced computational demand but also enhances the accuracy and efficiency of depth estimation, demonstrating the potential of FPN in 3D reconstruction tasks. (1)

$$\begin{array}{c} P_{i}=F_{i}\left(C_{i}\right)+\sum_{j>i}U_{j}\left(P_{j}\right) \end{array}$$ (1)

*P*_*i*_ represents the feature map of the *i*-th layer, *C*_*i*_ represents the input feature map of the i-th layer, *F*_*i*_ represents the convolution operation of the *i*-th layer, and *U*_*j*_ represents the upsampling operation.

### Cost volume

#### Cost volume method

**Fig 2:**
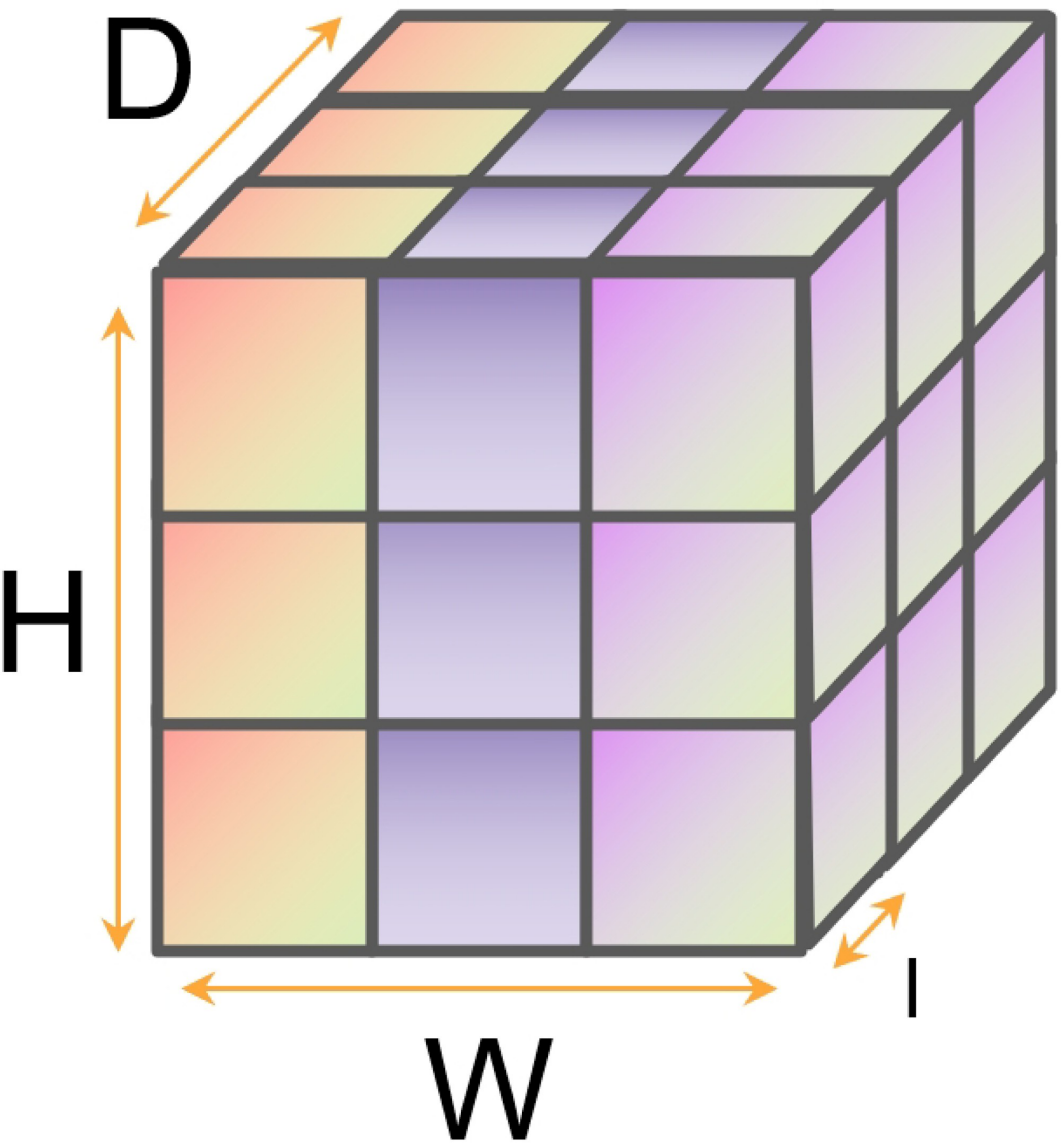
The form of the cost volume where *D* represents the number of hypothesized planes, *W* × *H* represents the spatial resolution, and *I* is the interval between planes.

In multi-view stereo (MVS), cutting-edge techniques have utilized deep learning methods to reconstruct scenes in an end-to-end manner. MVSNet [7, 10] proposed using forward parallel planes at different depths as hypothesis planes, typically determining the depth range through sparse reconstruction. Subsequently, the 2D feature maps are transformed onto the hypothesized planes of the reference camera using differentiable homography to form feature volumes, as shown in Fig 2. To integrate multiple feature volumes into a single cost volume, a variance-based cost metric was proposed to accommodate any number of input feature volumes. For the i-th view, the relationship between its feature map and the feature map of the reference view at depth *d* can be described by the homography *H*_*i*_(*d*), given by the following formula(2):

$$\begin{array}{c} H_{i}(d)=K_{i}\cdot R_{i}\cdot (I-\frac{(t_{1}-t_{i})\cdot n_{1}^{T}}{d})\cdot R_{1}^{T}\cdot K_{1}^{-1} \end{array}$$ (2)

Here, *K*_*i*_, *R*_*i*_, and *t*_*i*_ represent the intrinsic parameters, rotation, and translation of the i-th view’s camera, respectively, while *n*_1_ represents the principal axis of the reference camera. Then, by inverting *K*_*i*_ and *R*_*i*_, the features are mapped into the camera coordinate system. This mapping allows the feature map of the i-th view to be transformed under the coordinate system of the reference view.

#### Cascade cost volume

Existing MVS networks often use differentiable homography transformations to construct 3D cost volumes, mapping 2D feature maps to the hypothetical planes of the reference camera at each scale to form feature volumes. To integrate multiple feature volumes into a single cost volume, a variance-based cost metric is proposed to accommodate an arbitrary number of input feature volumes. This method is similar to traditional plane sweep stereoscopy, and the depth range is usually determined by sparse reconstruction. Fig 2 shows a cost volume; the standard resolution of a cost volume is defined as *W* × *H* × *D* × *F*, where *W* × *H* represents spatial resolution, *D* is the number of plane hypotheses, and F is the number of feature map channels. Increasing the number of plane hypotheses *D*, a larger spatial resolution *W* × *H*, and a finer plane interval can potentially enhance reconstruction accuracy.

Based on the previously predicted narrow depth range, a formula for cascaded cost volume is used. If we have a network estimating depths from coarse to fine, we can use the coarse prediction as a prior for the next stage, searching for hypothesis planes only within its neighborhood. This controls the size of the cost volume and significantly reduces memory requirements. A drawback of this method is its heavy reliance on the coarsest depth estimate. If the coarsest estimate is too far from the actual depth, the network fails to predict the correct depth, leading to significant errors, especially at object boundaries where the coarsest depth is often ambiguous. However, if we later merge multiple depth predictions, this issue does not persist because inconsistent predictions are not retained. Using a variance-based cost metric, multiple feature volumes are aggregated(3):

$$\begin{array}{c} C=\frac{\sum_{i=1}^{N}(F_{i}-\bar{F})}{N} \end{array}$$ (3)

For each feature volume *F*_*i*_, we calculate the square of its deviation from the mean of all feature volumes *F*, and sum these squared deviations. This sum is then divided by the number of feature volumes *N* to obtain the final cost volume *C*. This cost volume *C* reflects the consistency of features for each pixel point across different viewpoints: the smaller the variance, the more consistent the features across different views, which is considered to indicate a higher match quality for that pixel point.

Our method initially maps the source image features to the reference view coordinate system through a projection transformation matrix, thereby constructing the 3D cost volume. This process involves calculating the projection transformation, which is the transformation between the projection matrix of the source image camera and that of the reference view camera, followed by a transformation operation to resample the source image features.

For each predefined depth value, we use a meshgrid to generate a uniformly distributed grid and combine the internal and external parameters of the camera to perform a series of linear algebra operations. These operations transform the source feature maps spatially, reprojecting them onto various assumed depth planes. To ensure that this computation process does not interfere with gradient operations, we choose to perform these calculations in an environment that does not record gradients. The homography deformation function for stage *k* + 1 is as follows(4):

$$\begin{array}{c} H_{i}(d_{k}^{m}+\Delta_{k+1}^{m})=K_{i}\cdot R_{i}\cdot (I-\frac{(t_{1}-t_{i})\cdot n_{1}^{T}}{d_{k}^{m}+\Delta_{k+1}^{m}})\cdot R_{1}^{T}\cdot K_{1}^{-1} \end{array}$$ (4)

In this process, $d_{k}^{m}$ represents the predicted depth of the m-th pixel at stage k, while $\Delta_{k+1}^{m}$ is the depth residual at stage *k* + 1. Here, $H_{i}\left(d_{k}^{m}+\Delta_{k+1}^{m}\right)$ represents the homography transformation that adjusts the depth of the m-th pixel from the reference view to the i-th view. The homography matrix *H*_*i*_ is derived from a series of matrix operations, including the internal parameter matrix *H*_*i*_ and $K_{1}^{-1}$, the rotation matrices *R*_*i*_ and $R_{1}^{T}$, and the difference between the translation vectors *t*_1_−*t*_*i*_. Here, $n_{1}^{T}$ is the unit viewing direction vector of the reference camera. The entire expression, taking into account the depth adjustment $\left(d_{k}^{m}+\Delta_{k+1}^{m}\right)$, calculates the precise homographic transformation from the reference view to the i-th view.

The process primarily focuses on the mapping of pixel coordinates between consecutive stages. Specifically, we have defined an expression for mapping the m-th pixel’s coordinates in stage *k* + 1 in order to more accurately describe the disparity changes between pixels and their corresponding spatial relationships(5).

$$\begin{array}{c} C_{r}(d_{k}^{m}+\delta_{k+1}^{m})=x_{l}-(d_{k}^{m}+\delta_{k+1}^{m}) \end{array}$$ (5)

In this context, $d_{k}^{m}$ refers to the predicted disparity for the m-th pixel at the previous stage (i.e., stage *k*), which is derived from data and calculations from earlier stages and aims to provide a stable reference for subsequent processing. Meanwhile, $\delta_{k+1}^{m}$ represents the disparity residual that needs to be learned and adjusted for the same pixel point at stage *k* + 1. This residual value reflects the minor changes in pixel disparity from the previous stage to the current stage.

#### Multi-step depth enhancement refine

**Fig 3:**
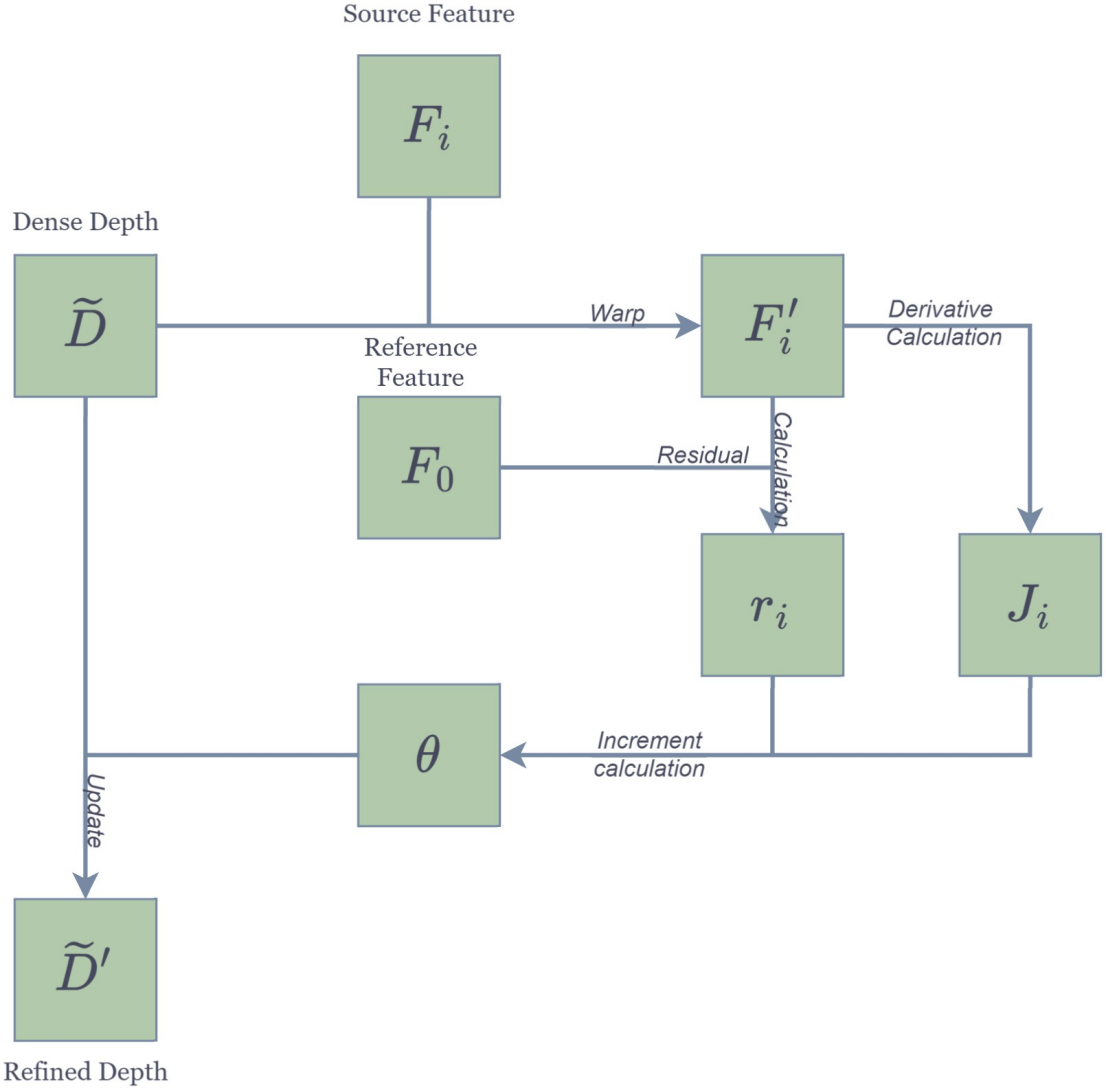
Structure of the depth optimization method.

Fig 3 illustrates our proposed depth optimization framework, which is one of the core components of our network. It aims to refine the initial dense depth map $\tilde{D}$ to enhance the accuracy of depth estimation, achieving a more detailed depth map ${\tilde{D}}^{\prime }$. The optimization process begins with the extraction of source features *F*_*i*_ from multi-view inputs, which are then warped to align with the features *F*_0_ of the reference view, resulting in aligned features $F_{i}^{\prime }$. A crucial step involves calculating the residuals *r*_*i*_ between the source and reference features, indicating the direction and extent of the current depth map correction.

Next, the derivative calculation module extracts the Jacobian matrix *J*_*i*_ from the residuals, which contains differential information about depth changes and forms the basis for the Increment Calculation process. This process uses the Jacobian matrix and residuals to compute the increments *θ* in depth adjustment, which directly indicate the extent of optimization applied to the dense depth map $\tilde{D}$. Through iterative application of these increments, the network gradually constructs a more refined depth map ${\tilde{D}}^{\prime }$.

This depth optimization process is crucial because it significantly enhances the accuracy of the final depth map. For a pixel *p* in the target image, with a depth value $\tilde{D_{p}}$, the error function guides the direction of depth optimization. In our network, depth optimization is an iterative process, with each step aimed at reducing the value of the error function and refining the current depth prediction. For a point *p* in the reference image, with depth $\tilde{D_{p}}$, the error function is(6):

$$\begin{array}{c} E(P)=\sum_{i=1}^{N}\|F_{i}(p_{i}^{\prime })-F_{0}(p){\|}_{2} \end{array}$$ (6)

Where *F*_*i*_ and *F*_0_ represent the depth positions of the original image and the reference image, respectively, $p_{i}^{\prime }$ is the reprojection of point *p*, and *F*_*i*_(*p*) is the feature at point *p* in the depth map of *F*_*i*_. The reprojection $p_{i}^{\prime }$ is calculated as follows(7):

$$\begin{array}{c} p_{i}^{\prime }=K_{i}(R_{i}R_{0}^{-1}(\tilde{D}(p)K_{0}^{-1}p-t_{0})+t_{i}) \end{array}$$ (7)

In the formula, ${\left\{K_{i},R_{i},t_{i}\right\}}_{\left(i=0\right)}^{N}$ represent the camera’s intrinsic parameters, the projection, rotation, and translation of the image, respectively. The multi-step depth refinement method aims to minimize *E*(*p*), starting from the initial depth ${\tilde{D}}_{p}$, and calculates the residual *r*_*i*_(*p*) for each image at point *p*(8):

$$\begin{array}{c} r_{i}(p)=F_{i}(p^{\prime })-F_{0}(p) \end{array}$$ (8)

Then, for each residual *r*_*i*_(*p*), we calculate their first derivative with respect to $\tilde{D^{\prime }}\left(p\right)$ as follows(9):

$$\begin{array}{c} J_{i}\left(p\right)=\frac{\partial F_{i}\left(p_{i}^{\prime }\right)}{\partial p_{i}^{\prime }}.\frac{\partial p_{i}^{\prime }}{\partial \;\tilde{D}\left(p\right)} \end{array}$$ (9)

Then, the increment *θ* for the depth is obtained as follows(10):

$$\begin{array}{c} \theta =-{\left(J^{T}J\right)}^{-1}J^{T}r \end{array}$$ (10)

Where *J* is the stack of Jacobian matrices ${J_{i}\left(p\right)}_{i=1}^{N}$, and *r* is the stack of residual vectors ${R_{i}\left(p\right)}_{i=1}^{N}$, thus the refined depth method is(11):

$$\begin{array}{c} {\tilde{D}}^{\prime }(P)=\tilde{D}(p)+\theta \end{array}$$ (11)

This method is differentiable, using multi-view image features, initial depth maps, and camera parameters as inputs to the optimization network, which then outputs refined depth maps. Typically, this method requires only one iteration update to quickly converge and does not necessitate complex sampling of depth hypotheses.

### Loss

We designed a comprehensive loss function encompassing multiple stages, where the total loss is computed as a weighted sum of the losses from each stage. The loss formula is as follows(12):

$$\begin{array}{c} TotalLoss=\sum_{k=1}^{N}\lambda_{k}L_{k} \end{array}$$ (12)

where *L*_*k*_ is the loss function for stage *k*, and λ_*k*_ are the weight parameters for each stage. The loss function for each stage is a function of the discrepancy between the estimated depth at that stage and the true depth, and can be expressed as(13):

$$\begin{array}{c} L_{k}=\frac{1}{|P_{\text{valid}}|}\sum_{p\in P_{\text{valid}}}\left|\tilde{D_{k}}\left(p\right)-\hat{D}\left(p\right)\right| \end{array}$$ (13)

Here, *P*_valid_ is the set of valid true depth points, $\tilde{D_{k}}\left(p\right)$ is the estimated depth value at pixel position *p* for stage *k*, and $\hat{D}\left(p\right)$ is the corresponding true depth value. For both initial depth estimation and refined depth estimation, an additional term is added to the loss function to balance the two(14):

$$\begin{array}{c} L_{k}^{\prime }=\frac{1}{|P_{\text{valid}}|}\sum_{p\in P_{\text{valid}}}(|\tilde{D_{k}}\left(p\right)-\hat{D}\left(p\right)|+\lambda |\tilde{D_{k}^{\prime }}\left(p\right)-\hat{D}\left(p\right)|) \end{array}$$ (14)

where $L_{k}^{\prime }$ represents the loss for the refined depth estimation at stage *k*, $\tilde{D_{k}^{\prime }}\left(p\right)$ is the refined depth estimation value for stage *k*, and λ is the weight parameter used to balance the initial and refined depth estimations, set to 1.0 in all experiments.

## Experiments

### DTU dataset

The DTU [20] dataset is a popular standard testing platform used for training and evaluating multi-view stereo (MVS) algorithms. This dataset comprises 80 meticulously set indoor scenes, each captured from multiple angles with 49 to 64 images. These images are equipped with precise intrinsic and extrinsic camera parameters, providing detailed perspective changes and rich disparity information. Designed to simulate various shooting conditions, including different lighting and backgrounds, the diversity of the DTU dataset makes it highly suitable for deep learning algorithms as it offers sufficient variation to train models’ generalization capabilities across different scenarios. Each scene not only includes high-quality color images but also provides high-precision ground truth depth maps obtained through structured light methods, which are crucial for training and validating the performance of MVS algorithms.

### Implementation details

We trained our method using the DTU dataset [20], setting the number of input images to N = 3 and implementing a two-level cost volume strategy, with the assumed depth layers set at 32 and 8, respectively. The corresponding depth intervals were set to 1x and 4x for each cascaded layer. The resolution of the images was fixed at 640x512. During the training phase, we employed the Adam optimizer, with hyperparameters *β*_1_ and *β*_2_ set to 0.9 and 0.999, respectively. The training lasted for 16 epochs, with the initial learning rate set at 0.001, and it was halved at the 10th, 12th, and 14th epochs to promote stability and convergence in the later stages of the model training.

### Results on the DTU dataset

The experimental evaluation of this study was conducted in an environment equipped with high-performance computing resources, utilizing an Intel(R) Xeon(R) Platinum 8255C CPU with 12 virtual CPU cores (at 2.50GHz) and an RTX 3090 graphics card with 24GB of VRAM. As shown in Table 1, we conducted a comparative analysis of several advanced multi-view stereo (MVS) reconstruction methods on the standard DTU dataset [20].

**Table 1:** Comparative analysis of multi-view stereo reconstruction methods on the DTU dataset.

Methods	Accuracy	Completeness	Overall
Camp [2]	0.835	0.554	0.965
Furu [31]	0.613	0.941	0.777
Tola [32]	0.342	1.19	0.766
SurfaceNet [9]	0.45	1.04	0.745
Point-MVSNet [21]	0.342	0.411	0.376
MVSNet [7]	0.456	0.646	0.551
PatchmatchNet [33]	0.427	0.277	0.352
CasMVSNet [22]	0.325	0.385	0.355
DSC-MVSNet [34]	0.316	0.372	0.344
Ours	0.386	0.305	0.3455

**Fig 4:**
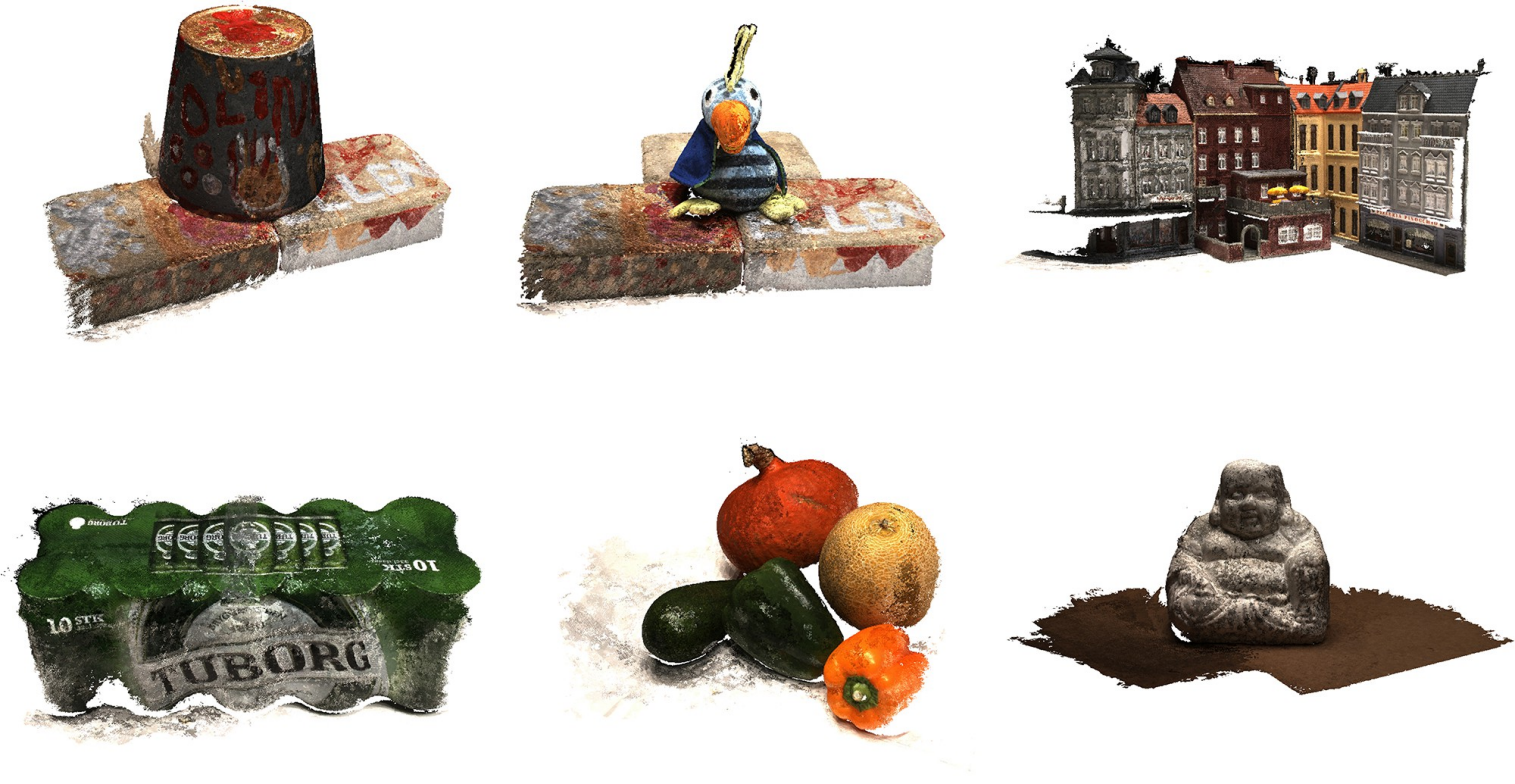
Multi-view stereo qualitative results of DTU dataset.

**Fig 5:**
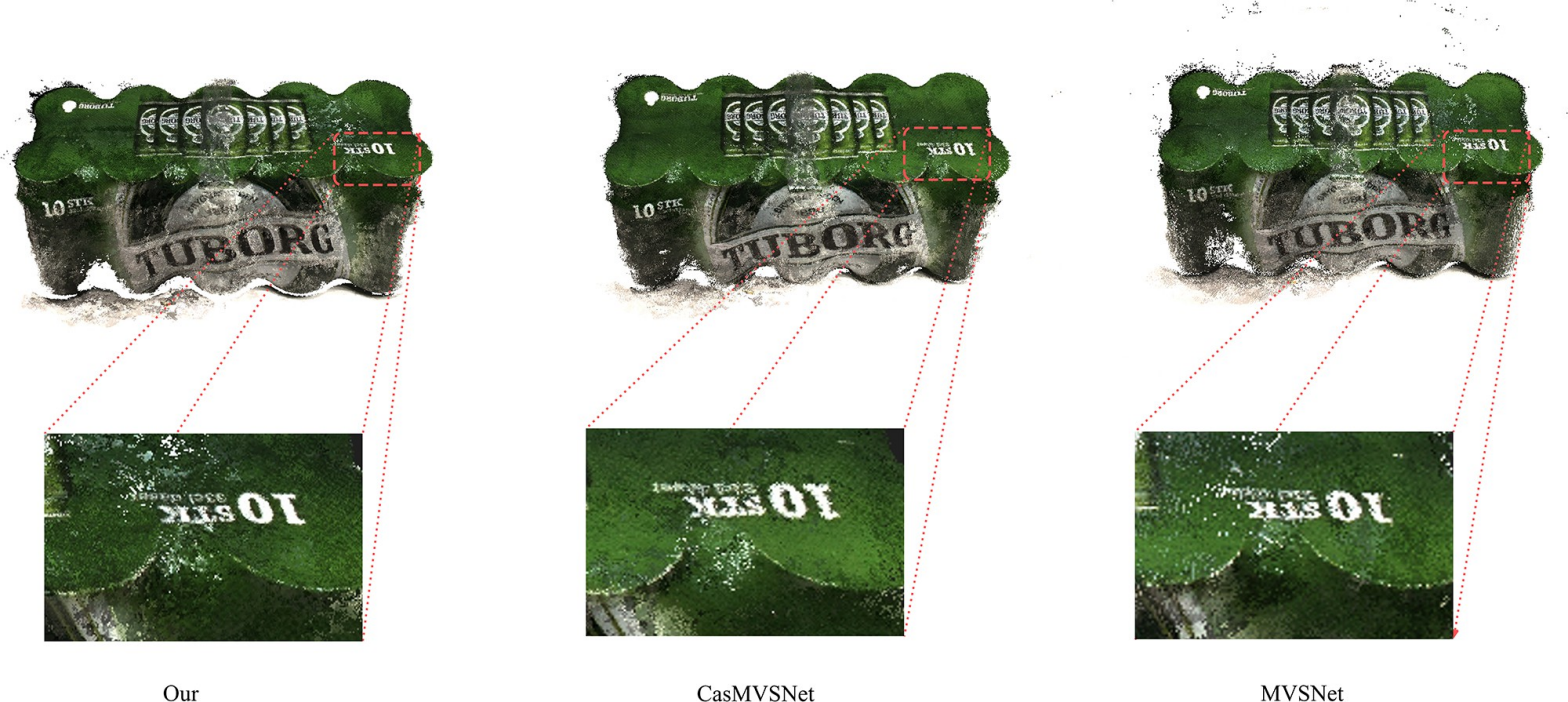
Comparison of reconstruction results on the DTU dataset. From left to right, the first column shows the results of our proposed method, the middle column shows the results of CasMVSNet [22], and the far right column shows the results of MVSNet. These magnified views allow for a comparison of the reconstruction details and text clarity between the different methods. Our method appears clearer and more detailed in its display of details, with text contours and backgrounds more distinctly separated.

The experimental results are shown in Fig 4. On three key performance indicators: accuracy (Accuracy), completeness (Completeness), and overall assessment (Overall), our method (Ours) achieved a completeness score of 0.305 and an overall score of 0.3455, demonstrating competitive performance. Notably, compared to our method, although CasMVSNet [22] showed a slight advantage in accuracy (Acc.) with a score of 0.325, our method exhibited significant improvements in both completeness (Comp.) and overall evaluation (Overall), as shown in Fig 5.

### Result on the Tanks and Temples dataset

**Fig 6:**
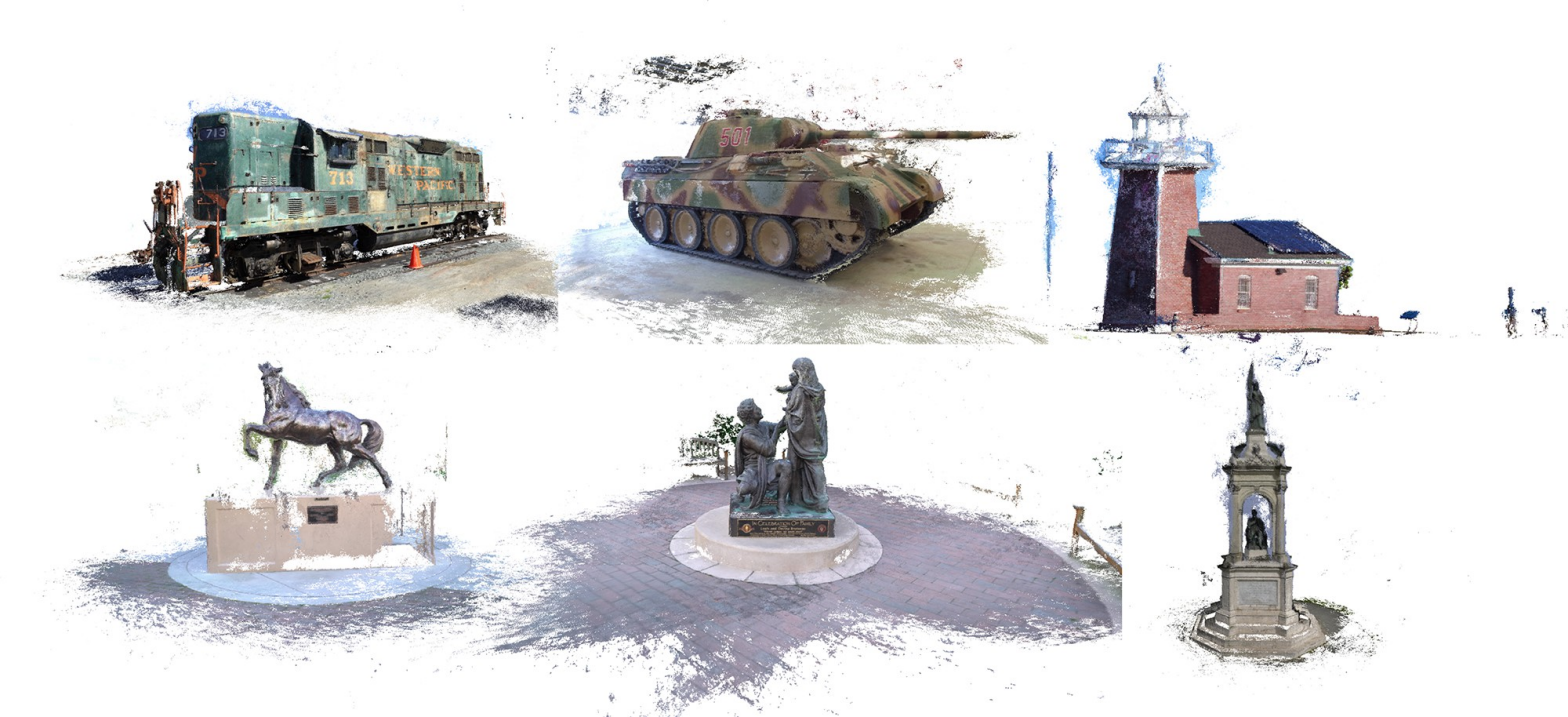
Multi-view stereo qualitative results of Tanks and Temples dataset.

The experimental results on the Tanks and Temples dataset [35], as shown in the Table 2, indicate that our Multi-Step Depth Enhancement Refine Network (MSDER-MVS) performed well across various scenes. In the Family scene, our method scored 77.26, higher than other methods such as CasMVSNet’s 76.36 and PatchmatchNet’s [33] 66.99. This demonstrates our method’s effective depth estimation capability when dealing with complex geometric structures. Additionally, in scenes like Lighthouse and M60, our method achieved scores of 58.49 and 59.85, respectively, surpassing other methods. In the M60 scene, our score exceeded CasMVSNet’s 56.11, indicating that the method can effectively handle challenges posed by different viewing angles and lighting conditions, demonstrating good generalization performance and stability. The result is demonstrated in Fig 6.

**Table 2:** Comparison of different MVS methods and their performance metrics, based on the Tanks and Temples dataset.

Method	Mean	Family	Francis	Horse	Lighthouse	M60	Panther	Playground	Train
COLMAP	42.14	50.14	22.25	25.63	56.43	44.83	46.97	48.53	42.04
R-MVSNet	48.4	69.96	46.65	32.59	42.95	51.88	48.8	52.00	42.38
Point-MVSNet	48.27	61.79	41.15	34.2	50.79	51.97	50.85	52.38	43.06
MVSNet	43.48	55.99	28.55	25.07	50.79	53.96	50.86	47.90	34.69
Fast-MVSNet	47.39	65.18	39.59	34.98	47.81	49.16	46.2	53.27	42.91
CasMVSNet	56.42	76.36	58.45	46.2	55.53	56.11	54.02	58.17	46.56
Ours	57.41	77.26	53.5	47.52	58.49	59.85	57.3	56.06	49.37

### Ablation experiment

To quantitatively analyze the contribution of each component of our proposed model to the final performance, we conducted detailed ablation experiments. The components evaluated include the number of depth layers, depth intervals, the dual-branch fusion architecture, depth optimization, and the multi-stage loss function. The evaluation metrics include accuracy (Acc), completeness (Comp), and overall performance (Overall), as shown in Table 3.

**Table 3:** Comparison of different MVS methods and their performance metrics, based on the DTU dataset.

Method	Depth Num.	Depth Interv.	Accuracy (Acc)	Completeness (Comp.)	Overall
MVSNet [7]	192	1	0.456	0.646	0.551
MVSNet+MSDER	192	1	0.449	0.512	0.480
Cas2MVSNet [22]	96, 96	2, 1	0.4352	0.4275	0.4314
CasMVSNet [22]	96, 48, 48	2, 2, 1	0.4479	0.4141	0.431
Ours (No Dual-Branch Fusion)	96, 96	2, 1	0.429	0.391	0.41
Ours (No Depth Optimization)	96, 96	2, 1	0.4	0.358	0.379
Ours (No Multi-Stage Loss)	96, 96	2, 1	0.409	0.351	0.38
Ours (Full Model)	96, 96	2, 1	0.385	0.305	0.344

MVSNet [7] set up 192 depth layers with a depth interval of 1 as the baseline model. Cas2MVSNet [22] adopted a dual-resolution strategy, setting 96 depth layers at each resolution with intervals of 2 and 1, aimed at enhancing the accuracy and completeness of depth estimation through two scales. CasMVSNet [22] further subdivided the depth layers into 96, 48, 48, with intervals of 2, 2, and 1, to explore the effects at a finer granularity.

Our method (Ours) improved upon the basis of Cas2MVSNet, maintaining the setting of 96, 96 depth layers with intervals of 2 and 1. Compared to Cas2MVSNet, our method showed advantages in all evaluation metrics. Particularly in accuracy (Acc), our method scored 0.429 for the configuration without the Dual-Branch Fusion, 0.4 for the configuration without Depth Optimization, and 0.409 for the configuration without Multi-Stage Loss. These scores demonstrate competitive performance against Cas2MVSNet’s score of 0.4352. Moreover, in overall performance (Overall), our full model configuration led with a score of 0.344, highlighting our method’s ability to balance accuracy and completeness.

Dual-Branch Fusion Architecture: To evaluate the impact of the dual-branch fusion architecture, we removed this component from the model and conducted experiments. The results, labeled as “Ours (No Dual-Branch Fusion)”, scored 0.41 overall. Depth Optimization: We disabled the depth optimization module to assess its contribution to the overall performance. The results of this experiment are shown as “Ours (No Depth Optimization)” and scored 0.379 overall. Multi-Stage Loss: To determine the effect of the multi-stage loss function, we conducted experiments without this component. The results are labeled as “Ours (No Multi-Stage Loss)” and scored 0.38 overall. These ablation studies provide a comprehensive understanding of the significance of each component in our proposed model, demonstrating that the full model configuration achieves the best performance across all evaluation metrics.

The ablation experiments not only confirmed the effectiveness of the proposed model in handling depth estimation tasks but also showed that managing depth layers meticulously and optimizing interval settings can enhance model performance. These results provide valuable insights into the application of deep neural networks in complex 3D vision tasks and validate the practicality and efficacy of our proposed improvement strategies in real-world scenarios.

## Conclusion

This paper introduces a novel Multi-Step Depth Enhancement Refine Network for multi-view stereo, which effectively enhances the quality of depth maps and optimizes computational efficiency through a dual-branch fusion architecture. With innovations in multi-scale feature extraction and cost volume construction, our method demonstrates superior performance in accuracy and completeness compared to existing technologies.

Experiments on the DTU standard dataset show that our network significantly improves the accuracy, completeness, and overall performance of depth prediction compared to other advanced MVS methods such as MVSNet [7], CasMVSNet [22], and Point-MVSNet [21]. Particularly, in the completeness (Comp.) and overall assessment (Overall) metrics, our method demonstrates its efficiency and accuracy with a considerable advantage. This achievement is attributed to our detailed study of details and careful design of the network structure, confirming the effectiveness of the multi-step depth refinement strategy in precise 3D reconstruction.

In summary, our research not only advances the application of deep learning in multi-view stereo matching tasks but also provides new perspectives and technological pathways for future 3D vision research. Although our method has achieved encouraging results, there are still challenges in realizing real-time applications and handling larger-scale datasets. Future work will focus on further optimizing the network architecture, enhancing the algorithm’s generalization capabilities, and exploring more efficient depth optimization techniques to facilitate deployment in a broader range of practical applications.

## Supporting information

S1 Table Detailed performance comparison on DTU data set. (XLSX)

S2 Table Detailed performance comparison on T&T data set. (XLSX)

S1 Raw images The original images in the article. (ZIP)
